# Task sharing and stepped referral model for community mental health promotion in low- and middle-income countries (LMIC): insights from a feasibility study in India

**DOI:** 10.1186/s40814-022-01159-0

**Published:** 2022-08-30

**Authors:** Saju Madavanakadu Devassy, Lorane Scaria, Natania Cheguvera

**Affiliations:** 1 Department of Social Work, Rajagiri College of Social Sciences (Autonomous), Rajagiri P.O, Kalamassery, Cochin, Kerala India; 2 Rajagiri International Centre for Consortium Research in Social care (ICRS), Rajagiri P.O, Kalamassery, Cochin, Kerala India; 3 Honorary Principal Fellow, Department of Social Work, Melbourne School of Health Sciences, University of Melbourne, Melbourne, VIC 3010 Australia

**Keywords:** Mental health, Lay mental health worker, Community mental health, Task sharing, Health referral, India

## Abstract

**Background:**

This study is a low-cost community mental health task-sharing model driven by university students to strengthen the mental health workforce in poor resource settings. This article presents the feasibility of a stepped referral model using the community health workforce and university students. The primary feasibility objective is to detect and refer people with mental illness from the community using a task-sharing approach.

**Methods:**

We tested the model using a cross-sectional, one-phase door-knock survey in three geographically defined locations in Kerala, India, between May and July 2019. Students surveyed 549 residents above 18 years of age who consented to participate in the study to detect depressive symptoms and suicidality. The feasibility of the current model was evaluated based on four criteria: (a) identification and deployment of untapped human resources, (b) coordination of community health resources, (c) the acceptability of stepped referral pathways, and (d) identification of implementation challenges.

**Results:**

The mean age of the participants was 38.8, and more than 62% of the respondents were women. The results showed that 11.29%, 8.38%, and 4.91% of people reported mild, moderate, and severe levels of depression, respectively, and suicidal thoughts were found in 6.9% and suicidal ideation in 1.8%. The odds of depression were higher among females compared to males (OR: 1.64 (0.75–2.52), poor people (OR: 2.01 (1.14–2.88), and people with chronic illnesses (OR: 2.03 (1.24–2.81). The agreement of the findings with professional-administered research validated the strategy’s efficiency. Twenty-seven patients with severe/extreme degrees of depression were sent for high-intensity interventions led by the mental health team, whereas 135 individuals with mild and above depression were referred for low-intensity interventions.

**Conclusions:**

The newly recruited mental health workforce-driven screenings were acceptable and effective in detecting mental illness in the community population. We tested the care coordination systems and processes in creating referral pathways for the detected patients. Further, task-sharing stepped referral model will be tested in five panchayats (the lowest tier of local self-government) before replicating the model across India through Unnat Bharat Abhiyan (UBA) scheme.

## Key messages regarding the feasibility

What uncertainties existed regarding the feasibility?

- Convincing the mental health professionals, community health workers, and community population about the task-sharing stepped referral model and getting it accepted by all was the major challenge.

What are the key feasibility findings?

- The study results were comparable with existing mental health professional-driven prevalence studies, especially on depression and suicidality, and could enumerate the profile of high-risk groups cost-effectively. The university students and community volunteers are capable to implement the stepped referral model provided they receive systematic training on the standard operating procedures and referral pathway manuals.

What are the implications of the feasibility findings for the design of the main study?

- The proposed stepped referral model can potentially optimize scarce professional mental health resources.
- Study evidenced the feasibility of task sharing stepped referral model that can be replicated across India through the UBA scheme.

## Background

The gap between individuals who need and receive mental health care is enormous worldwide [1] and is close to 85% in India [2]. Further, 1 in every 7 Indians is affected by mental disorders of varying severity [3]. Low mental health literacy [4] and high reliance on traditional healers due to cultural and spiritual interpretations of mental illnesses [5, 6] add to the problem severity. Consequently, more than 75% of individuals who would benefit from treatment are left undetected and untreated [7].

Previous studies emphasized recognizing the warning signs [7] and introducing preventive measures for high-risk populations and circumstances [8] as critical in community mental health. The two major building blocks to improving community mental health outcomes are service delivery and the availability of the health workforce [9]. In India, the number of qualified mental health professionals is disproportionate such that it is practically impossible to address the mental health needs of everyone [10]. Only 10% of individuals with a mental illness will be attended to, even if all the professionals worked full time [11].

Strategies to address mental health human resources scarcity [12] requires socially sensitive educated people with the capacity to carry out the newly shared tasks with an additional training and supervision by the professionals. Though the WHO mhGAP action plans suggested the task shifting and task sharing as an effective strategy to scale up the mental health services to all [13], seldom these strategies are tested for its acceptance, accuracy, and effectiveness.

The study was done as part of Unnat Bharat Abhiyan (UBA), a programme initiated by the Ministry of Human Resource Development (MHRD), Government of India, to enable university and college students to work in rural areas to identify and provide solutions to the development challenges. This program can reach 18.7 million people nationwide through 2476 participating Universities. This stepped referral model aims to share the task to screen and provide referral services for community population, detected with depression and suicidality to appropriate mental health services [14], which is first of this kind in India.

This stepped referral model is modelled after the stepped-care model [15], where the University students joined hands with Accredited Social Health Activists (ASHAs) to implement the current project. ASHA workers are defined as lay health workers selected from the village, each catering to an average of 1000 population. Their primary role is to create health awareness and its social determinants, community mobilization for local health planning, and increased utilization of the existing health services. The ASHAs were included to utilize their domicile advantage in the community to get easy access and acceptability [16]. Youth enrolled in any undergraduate or graduate programme the implementing college offered were volunteers of this model. The feasibility of linking the already-existing community health infrastructure with the academic community, which is controlled by coordination systems and protocols for detection and stepped-up referral pathways, has not yet been investigated.

Accumulating evidence suggests that task-shifting approaches with the non-specialist health professionals and lay workers with brief training and appropriate supervision by mental health specialists are sufficient to detect, diagnose, and refer individuals with mental disorders in the community [15]. Although the task sharing with student volunteers, faculty, and ASHAs was not rigorously examined, if it was found to be successful, it might help India’s shortage of mental health professionals.

## Materials and methods

The study introduced the task-shifting model for mental health community screening and stepped referral using an untapped mental health workforce of ASHAs, academic students, and faculty. The study included a one-phase, community survey to identify and refer mental illness in the community. The objectives, outcome assessment, and the measures for evaluating the feasibility objectives for the study are described in Table 1.

**Table 1:** Feasibility objectives, outcome, and indicators

Objectives	Outcome assessor
To generate a continuum of collaboration between community health infrastructure, university faculty, and student volunteers to screen, detect, and refer the people with mental illness	Human resource identification, Memorandum of Understanding (MoU) between the institutions to collaborate, and developing linkages with mental health service providers to establish referral pathways
To train the student volunteers and ASHAs to screen, diagnose, recommend appropriate stepped referral pathways, and introduce supervisory systems and processes	The competency for accurate diagnose and ability to recommend the detected patients to appropriate referral services
To test the accuracy and acceptability of community screening	Comparability of study results with similar studies and number of detected people accepting the referral recommendations

### Study participants and study designs

**Fig. 1:**
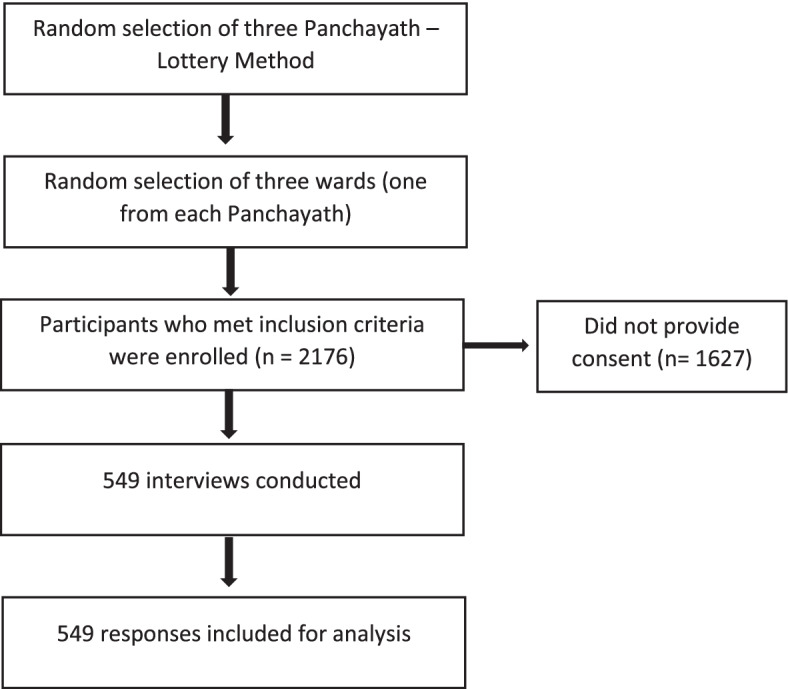
Data collection process

A cross-sectional one-phase community survey was conducted among 549 residents in geographically defined catchment areas in Ernakulam District, Kerala, India, between May 2019 and July 2019. Three local self-government (LSG) divisions, Chottanikkara, Vazhakulam, and Kalamassery, were randomly selected through the lottery method. One ward each (a ward is a subdivision of LSG with clear geographical boundaries and an average of 800 to 900 adults) from these three panchayats (a panchayat is a local self-government body comprising a number of wards) was randomly selected for data collection. Of 2176 eligible participants, 549 consented (25.23%) to be part of the study. We chose a door knock survey method as it proven effective for data collection [17]. However, a relatively low response rate of 25% could be attributed to prevailing social stigma related to psychiatric disorders and the reluctance to accept the students screening them for mental disorders. Additionally, many potential participants were unavailable at home due to their job or other social and cultural commitments. Eligibility criteria included the individuals aged ≥ 18 years, resident in this locality for a minimum of 1 year, who consented to participate. Participant recruitment is described in detail in Fig. 1.

**Fig. 2:**
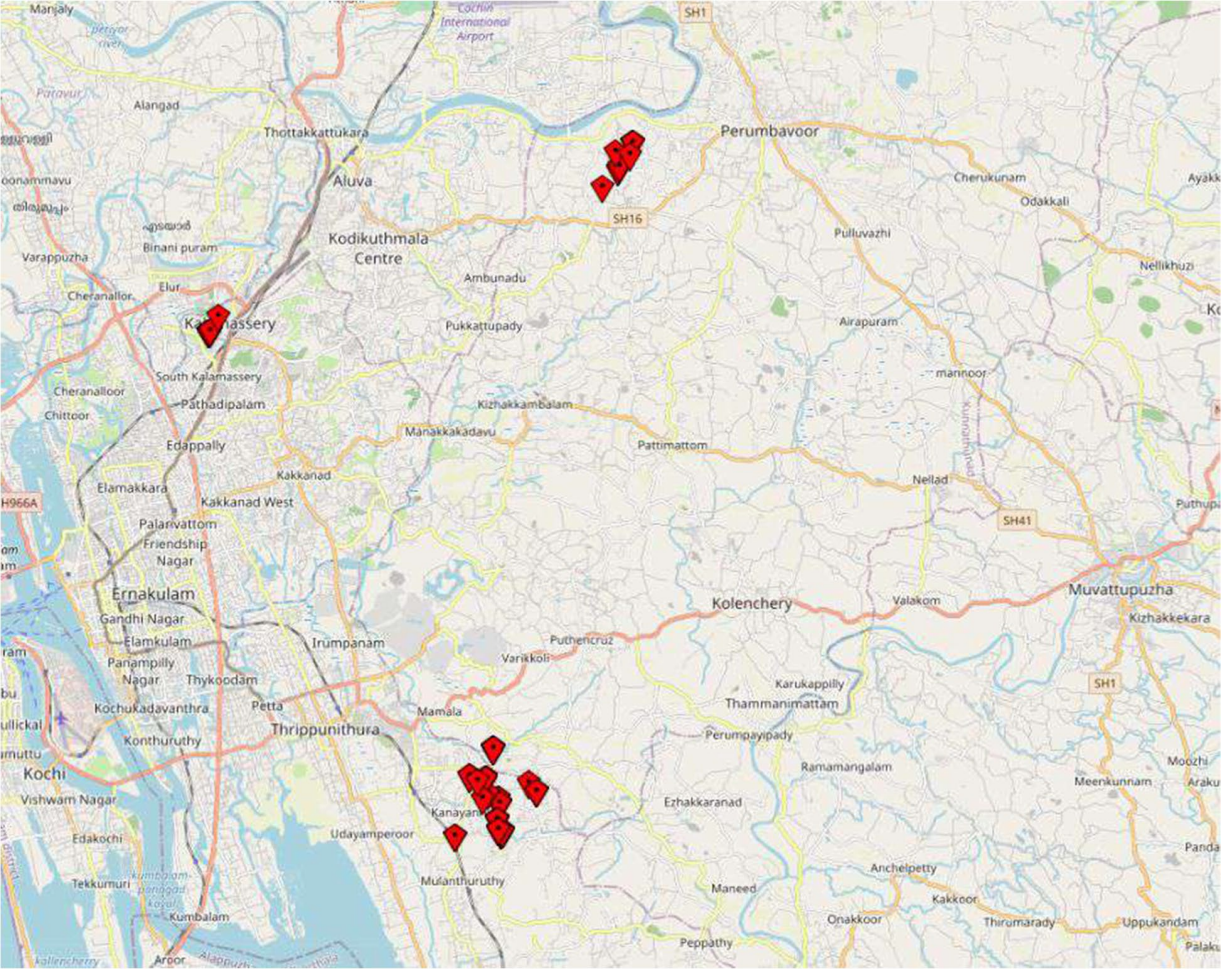
Geospatial representation of the respondents

The trained University students collected data from participants’ homes. An indigenously developed smartphone-based application was used to collect the data and record geospatial details (Fig. 2). The details of each household containing names, addresses, ID numbers, contact details, and other information were stored on secure servers.

### Data collection

We obtained informed consent after explaining the purpose, nature of the interview, possible benefits and risks of participation, confidentiality, and the participant’s right to withdraw from the study. The participant assessment lasted for approximately 15 min.

### Training and supervision

As a prelude to data collection, initial training was given to the students on general themes of mental health, interviewing skills, study instruments, and referral pathways to ensure protocol fidelity. The recruited students further underwent structured 2 days of training on mental health screening, referral, and promotional activities. The primary focus of the training was on the administration of the tools and their critical values to detect caseness among participants. Then, they got familiarized with the psychoeducational brochures that contained the cardinal symptoms and early warning signs, self-care materials including, exercises, social networking options, information about the mental health services in their locality, and referral pathways. Role plays and mock interviews were performed to familiarise the students with the assessment and referral process. The academic faculty periodically monitored the students, who acted as mentors and supervisors in the process. A similar approach of data collection was proven effective for community based mental health screening in India [18].

### Task shifting and stepped referral model of mental health promotion

The stepped referral model implemented through the students in the community proceeded through multiple phases. The initial phase involved community-based assessment and prevention activities where the students handed over the brochures and leaflets with self-care-related psychoeducation content to every household after explaining the content as a primary prevention strategy. Following the assessment, the second phase involved referral of participants with mental health symptoms. The screening results were communicated to the person detected positive and his or her family. Participants with suicide thoughts or attempts were immediately referred to psychiatrists for urgent care. Participants with mild and above levels of depression symptoms were advised to get psychosocial support from the family counselling centres (FCC- funded by the State Social Welfare Board, Government of Kerala) located in their locality. Cases with severe psychiatric symptoms were referred to the mental health facility after a comprehensive assessment by the professional counsellor working in the family counselling centres. These assessments were handed over to the psychiatrists for further treatment. They also provided psychoeducation materials to those who screened positive for depression and suicidality. The lay health resource was the liaison between the detected client and the appropriate referral services. People with adverse life experiences, such as any family member with alcoholism, loss, grief, trauma, disputes, and living alone, were linked with the ASHA workers and to family counselling centres for follow-up and intervention.

### Measures

Culturally validated assessments of participants’ socio-demographic profile, physical and mental health, and non-communicable disease risk factors were combined to develop the dataset. All study instruments were translated, back-translated, and assessed for acceptability and conceptual equivalence. The translations were carried out locally by professionals fluent in English (the language of the instruments) and Malayalam (the local language), which a language expert further reviewed before its use for data collection.

### Socio-demographic questionnaire

The socio-demographic questionnaire had age, gender, economic status, education, occupation, marital status, religious affiliations, self-reported hypertension, and diabetes. The gender was categorized as male or female. Economic status was ascertained as above poverty line (APL) and below poverty line (BPL). The education level was shown and coded as follows: not literate, up to class 10th, class 12th, and graduate/diploma and postgraduate. The occupation was determined as unemployed, farming/fishing in their land, daily wage jobs, salaried employment, housewife/husband, and other business. The marital level was recorded as unmarried, married, widowed, and divorced/separated. The caste category was described as general and SC/ST/OBC. Finally, the questionnaire also registered the self-reported chronic health conditions, such as diabetes and hypertension.

### Patient health questionnaire-9 (PHQ-9)

PHQ-9 was administered to assess depression levels in the study population. It scored each of the nine Diagnostic and Statistical Manual of Mental Disorders (DSM-V) criteria on ‘0’ (not at all) to ‘3’ (nearly every day). The total scores range between 0 and 27, with 0 representing the lowest possible scores of depressive symptoms and 27 representing the highest score. The internal consistency reliability (Cronbach’s alpha 0.89) and interrater reliability (interclass correlation coefficient, 0.94; 95% confidence interval (CI): 0.86–0.95) were high. This assessment tool was validated in India, with a positive likelihood ratio of 8.3 [19].

### Suicide risk assessment

The suicidality risk was assessed based on suicidal thoughts and suicidal ideation. The following questions were asked to measure suicide risk among the population. ‘Have you thought about being dead or what it would be like to be dead? Have you wished you were dead or wished you could go to sleep and never wake up? Do you wish you weren’t alive anymore? Have you thought about doing something to make yourself not alive anymore? Have you had any thoughts about killing yourself?’

### Statistical analysis

Sample size was calculated using the formulae, *n* = *Z*^2^*P* (1-*P*)/d^2^ [20], where in *Z*, the standard normal variate is 1.96 at 5% type 1 error (*p* < 0.05); *P*, the expected proportion in population based on previous studies is 0.27 [21], and *d*, the precision is estimated at 5%. The calculated sample size is 176 from each ward making the total estimated sample size 528. All statistical analyses were performed using the STATA-14 software. We performed descriptive analysis to find depression and suicidal ideation prevalence among people from different demographic profiles. Chi-square tests compared the levels of depression with suicidality, gender, and socioeconomic status. We performed linear regression analysis to evaluate the variables associated with depression and calculated a 95% CI. *P*-value < 0.05 indicates statistical significance. The association between suicidality and depression was determined using multinomial logistic regression analysis.

## Results

### Demographic characteristics of recruited participants

The mean age of the participants was 38.88 ± 16.09 (standard deviation, SD) years. Among the participants, 11.29%, 8.38%, and 4.91% reported mild, moderate, and severe levels of depression, respectively, as measured by PHQ-9. Suicidal thoughts were found in 6.9% of the total study population and suicidal ideation in 1.8%. One hundred thirty-five respondents with mild and above depression were referred to FCC centres. Among the people who got referred to counselling centres, 104 of them were females, 77 of them were below 45 years of age, and 89 were married.

The highest risk of suicide implied the existence of both thoughts and ideation, and the prevalence of depression was significantly associated with suicidal risk in the population. Further, females from lower socioeconomic backgrounds are likely to experience high depression and suicidality compared to their male counterparts. In the current study, those with severe and extreme levels of depression had a higher risk for suicidality. The level of depression was higher in females and people from lower socioeconomic status. Among the respondents, 11% of females exhibited moderate levels of depression, and 4.6% showed severe/extreme depression. Furthermore, 9.5% in the BPL category had moderate levels of depression, and 5.9% had severe levels of depression. Thus, almost 90% of the people with a high risk for suicidality belonged to moderate and severe depression groups (Table 1).

Table 2 represents the association of different levels of depression with chronic conditions and demographic variables of gender and poverty. Gender and socioeconomic status are significant factors associated with depression and suicidality in this population. In addition, moderate, severe, and extreme levels of depression were associated with self-reported diabetes and hypertension, and the difference was statistically significant. Among individuals with self-reported diabetes, almost 37% had mild and above depression, while 54% had mild and above depression among those with self-reported hypertension (Table 3).

**Table 2:** Rates of depression and suicidality among groups with different socio-demographics and chronic health conditions. *N* = 549

Variables	Subject	Presence of depression	Presence of suicidal thoughts	Presence of suicidal ideation
**Total**		**135 (24.59%)**	**38 (6.92%)**	**10 (1.82%)**
**Gender**				
Male	205 (37.34%)	31 (15.12%)	4 (1.95%)	1 (0.49%)
Female	344 (62.66%)	**104 (30.23%)**	**34 (9.88%)**	9 (2.62%)
**Age**				
≤ 24	112 (20.40%)	26 (23.21%)	5 (4.46%)	0
25–44	265 (48.27%)	51 (19.25%)	17 (6.42%)	6 (2.26%)
45–64	120 (21.86%)	32 (26.67%)	13 (10.83%)	4 (3.33%)
≥ 65	52 (9.47%)	**26 (50.00%)**	3 (5.77%)	0
**Poverty**				
APL	324 (59.45%)	61 (18.83%)	17 (5.18%)	5 (1.52%)
BPL	221 (40.55%)	**71 (32.13%)**	**21 (9.5%)**	5 (2.26%)
**Education**				
Not literate	12 (2.21%)	**5 (41.67%)**	1 (8.33%)	0
Did not complete primary education	125 (22.77%)	47 (37.60%)	12 (9.60%)	4 (3.2%)
Completed primary education	156 (28.42%)	38 (24.36%)	14 (8.97%)	2 (1.28%)
Completed secondary education	93 (17.10%)	21 (22.58%)	5 (5.38%)	2 (2.15%)
Completed tertiary—graduate/diploma	125 (22.98%)	21 (16.80%)	6 (4.80%)	2 (1.60%)
Completed tertiary—postgraduate	38 (6.99%)	3 (7.89%)	0	0
**Occupation**				
Unemployed	127 (23.13%)	33 (25.98%)	6 (4.72 %)	0
Farming/fishing, etc., in own land	196 (35.70%)	63 (32.14%)	14 (7.14 %)	3 (1.53 %)
Daily wage jobs	104 (18.94%)	24 (23.08%)	8 (7.69 %)	4 (3.85 %)
Salaried employment	104 (18.94%)	13 (12.50%)	9(8.65 %)	3 (2.88 %)
Other business	18 (3.28%)	2 (11.11%)	1 (5.56 %)	0
**Marital status**				
Unmarried	126 (23.16%)	27 (21.43%)	6 (4.76%)	0
Married	385 (70.13%)	89 (23.12%)	29 (7.53%)	9 (2.34%)
Widowed	32 (5.83%)	16 (50.00%)	2 (6.25%)	1 (3.13%)
Divorced/separated	6 (1.09%)	3 (50.00%)	1 (16.67%)	0
**Category**				
General	160 (29.14%)	27 (16.88%)	8(5%)	1 (0.63%)
SC/ST/OBC	343 (68.19%)	108 (27.76%)	30 (7.71%)	9 (2.31%)
**Presence of HTN**^**a**^	75 (13.66%)	40 (53.33%)	11 (14.67%)	5 (6.67%)
**Presence of DM**^**b**^	69 (12.57%)	25 (36.23%)	3 (4.35%)	0

^a^HTN-self-reported hypertension

^b^DM-self-reported diabetes

**Table 3:** Chronic illness and depressive symptoms

	Chronic conditions				Demographics			
Variables	Self-reported diabetes		Self-reported hypertension		Gender		Poverty level	
Depression	***Yes (n=69)***	***No (n=480)***	***Yes (n=75)***	***No (n=474)***	***Male***	***Female***	***APL***	***BPL***
Normal	44 (63.77%)	370 (77.08%)	35 (46.67%)	379 (79.96%)	174 (84.9%)	240 (69.8%)	264 (80.5%)	150 (67.9%)
Mild	15 (21.74%)	47 (9.79%)	19 (25.33%)	43 (9.07%)	18 (8.8%)	44 (12.8%)	34 (10.4%)	28 (12.7%)
Moderate	4 (5.80%)	42 (8.75%)	9 (12.00%)	37 (7.81%)	8 (3.9%)	38 (11%)	25 (7.6%)	21 (9.5%)
Severe	3 (4.35%)	13 (2.71%)	9 (12.00%)	7 (1.48%)	1 (0.5%)	15 (4.4%)	3 (0.9%)	13 (5.9%)
Extreme	3 (4.35%)	8 (1.67%)	3 (4.00%)	8 (1.69%)	4 (0.2%)	7 (0.2%)	2 (0.6%)	9 (4.1%)

Table 4 elaborates the regression analysis of depression and various socio-demographic characteristics and chronic disease conditions among the participants. Depression was two-fold more likely for patients with a self-reported diagnosis of diabetes and hypertension (*B* = 2.03, *P* = 0.000). In addition, people from the BPL group were more likely to have depressive symptoms (*B* = 2.01, *P* < 0.001) than those in the APL group. The associations remained statistically significant even after adjusting for age among the participants.

**Table 4:** Factors associated with depression: linear regression analysis

Variables	Unadjusted ***B*** (95% CI), ***P*** value	Adjusted B* (95% CI), ***P*** value
Gender—female	1.64 (0.75–2.52)	1.73 (0.85–2.61)
Age	0.04 (0.01–0.07)	
Poverty-BPL	2.01 (1.14–2.88)	1.93 (1.06–2.79)
Education	− 0.47 (− 0.68 to − 0.28)	− 0.41 (− 0.64 to − 0.17)
Chronic disease comorbidity	2.03 (1.24–2.81)	1.87 (1.00–2.74)

In the current study, suicidality was measured in terms of suicidal ideation and suicidal thoughts and was associated with depression (Table 5). Table 5 describes the multinomial regression analysis of depression and suicidality. Participants in the medium-risk group were 11 times more likely to have depression, while participants in the high-risk group had 34 times more chance of depression.

**Table 5:** Multinomial logistic regression analysis of suicidality with depression

Suicidality and depression	Relative risk ratio (95% CI), ***P*** value
Suicidality—no risk	1ref
Suicidality—medium risk	11.6 (4.8–28.02
Suicidality—high risk	34.8 (4.4–277.7)

### Effectiveness of the model proposed

Overall identified prevalence of depression from the community dwelling adults is 25% which is similar to other studies conducted in a similar setting through trained professionals which accounts for the accuracy of the task shifting model proposed [21]. The task shifting for community screening was performed using a mental health team of 30 selected students, 4 faculties, and 6 ASHA workers (2 for each ward). Furthermore, a continued collaboration was set up with the panchayat functionaries by the academic institutions as part of the programme to ensure continued support and referral of mental health problems in the community.

## Discussion

Availability and scale-up of essential mental health services to achieve universal coverage of mental health are often impeded due to mental health workforce shortages [22]. It is particularly true in LMICs, where the proportion of mental health professionals and the population is vast; WHO estimates that 1.18 million mental health workers need to close the mental health treatment gap [23]. The educated youth is an untapped human resource, with additional training to take up the civil society leadership in mental health.

The primary aim of this study was to test the feasibility of a college student-driven community mental health task-sharing model to increase mental health care coverage in LMICs. The results of the community screening were consistent with India’s other prevalence studies. For example, the National Mental Health Survey reported a prevalence of 10.78% for suicide [24], while our research detected suicidal thoughts in 7.18% and suicidal ideation in 1.89% study population; thus, a total of 9.07% of people had a prevalence of suicide. The current study identified aged, widowed, or separated females with low socioeconomic status, fewer years of education, and one or more chronic conditions are most vulnerable to depression. Previous evidence also highlighted that depression is significantly associated with old age [25], female gender [26], poverty [27], and chronic conditions of hypertension and diabetes [28]. Our study was consistent with the western pattern of the high prevalence of suicidal behaviour among females in the 45–64 age group. This phenomenon could be attributed to Kerala’s high human development indices comparable to developed countries [29]. The collaborative stepped referral model used in this study confirms the relative accuracy of the screening procedure and referral pathways in the real-world scenario. Lay health workers-led screening was feasible due to the brevity of the instruments used in the initial screening and the training on the newly assigned tasks. This stepped-referral approach facilitates the optimum use of scarce professional resources.

Effective task-sharing requires the system to work in harmony through better functional and geographical coordination, adequate supervision, robust tools and resources, quality training [30], and successful redistribution of tasks among ASHA workers and the university student teams. Acceptability of task-sharing interventions by the stakeholders, both community and health system personnel, is also critical to the success of the task-sharing model.

Four out of the six building blocks of health system delivery, service delivery, health workforce, information technology, and leadership [9] are effectively integrated into this model. Service delivery is within the community mental health framework, where the student teams are supervised by the mental health team and supported by the ASHAs. Information technology was used to collect, store, and manage the data and record the participants' geospatial details for locating them for future referrals. The use of technology was advantageous, as it helped scale up this model without additional stationery or material cost. Moreover, it facilitates big data management for storage and comparisons for health planning. Finally, this stepped referral model reduces professionals’ caseload, enabling them to spend the scarce resources judiciously to increase mental health coverage without compromising the quality.

If this task sharing is implemented all over India, it can address the most critical mental health barrier of inadequate mental health human resources. Additionally, this is a practical step to address the rural-urban divide in distributing mental health facilities, depriving the rural population of getting their rightful slice. Further, this will help deal with the huge treatment gap, poor health illiteracy [31], socio-cultural stigma, and discrimination [32]. Additional to this program’s early detection and treatment advantage, it provides mental health literacy to the community. Literature evidence shows that mental health awareness significantly improves mental health literacy and professional help-seeking behaviour and reduces social stigma [33].

## Strengths and limitations

This study is not without limitations; students’ limited prior experience of community surveys is addressed through various interactive and engagement strategies. Despite the relatively small sample size and the poor response rate, the findings were in agreement with the results of large sample studies, demonstrating this study’s unbiases. Social desirability bias among participants is possible due to stigma related to mental health, leading to underreporting of their suicidal thoughts/ideations/attempts.

## Conclusions

The efficiency and effectiveness of student-led mental health detection and stepped referral model is a compelling reason to incorporate mental health as a priority area of the UBA programme to scale up the mental health services to the community cost-effectively.

## Data Availability

The data that supports the findings of the paper is available from the corresponding author upon reasonable request.

## Abbreviations

UBA: Unnat Bharat Abhiyan (UBA)
MHRD: Ministry of Human Resource Development
ASHAs: Accredited Social Health Activists
APL: Above poverty line
BPL: Below poverty line
DSM: Diagnostic and Statistical Manual of Mental Disorders
CI: Confidence interval
